# Fabrication and Robotization of Ultrasensitive Plasmonic Nanosensors for Molecule Detection with Raman Scattering

**DOI:** 10.3390/s150510422

**Published:** 2015-05-04

**Authors:** Xiaobin Xu, Kwanoh Kim, Chao Liu, Donglei Fan

**Affiliations:** 1 Materials Science and Engineering Program, the University of Texas at Austin, Austin, TX 78712, USA; E-Mails: xxu.uta@gmail.com (X.X.); chaoliu2011@utexas.edu (C.L.); 2 Department of Mechanical Engineering, the University of Texas at Austin, Austin, TX 78712, USA; E-Mail: kkim@austin.utexas.edu

**Keywords:** SERS, sensors, NEMS, nanomotors, drug delivery, manipulation

## Abstract

In this work, we introduce the history and mechanisms of surface enhanced Raman scattering (SERS), discuss various techniques for fabrication of state-of-the-art SERS substrates, and review recent work on robotizing plasmonic nanoparticles, especially, the efforts we made on fabrication, characterization, and robotization of Raman nanosensors by design. Our nanosensors, consisting of tri-layer nanocapsule structures, are ultrasensitive, well reproducible, and can be robotized by either electric or magnetic tweezers. Three applications using such SERS nanosensors were demonstrated, including location predictable detection, single-cell bioanalysis, and tunable molecule release and monitoring. The integration of SERS and nanoelectromechanical system (NEMS) devices is innovative in both device concept and fabrication, and could potentially inspire a new device scheme for various bio-relevant applications.

## 1. Introduction 

### 1.1. The Discovery and History of Surface Enhanced Raman Scattering

It has been over 40 years since in 1973 Fleischmann *et al.*, first observed the substantial enhancement of the Raman scattering spectrum of sub-monolayer pyridine molecules on electrochemically roughened silver (Ag) electrodes [1]. Four years later, in 1977, two research groups independently reported that the substantial enhancement of Raman scattering could not be understood either by the concentration increment of molecular species or by the increased surface area due to the electrochemically roughened process [2,3]. Jeanmaire and Van Duyne pioneerly proposed that the enhancement is due to the enhanced localized electric (*E*-) fields in closely positioned Ag nanoparticles, the so-call hot spots [2], while Albrecht and Creighton suggested that the enhancement is due to charge transfer between plasmonic nanoparticles and analyte chemicals [3]. Both theories are proved and widely accepted today and this phenomenon is now known as surface enhanced Raman scattering (SERS) [4,5].

The investigation of SERS in the early years has been largely focused on phenomena occurring on electrochemically roughened Ag substrates. However, electrochemically roughened Ag substrates cannot provide significant SERS enhancement for single-molecule detection. Until the recent decades, the design and synthesis of SERS substrates have been remarkably improved, largely due to the vigorous advances in micro- and nanofabrication. An enhancement factor (EF) of ~10^10^ and above has been reported, which can readily detect single molecule of various species [6,7]. Also owing to the advantage in determining molecules in a label-free and multiplex manner, SERS was applied for detection of various bio-relevant species, such as DNA/gene [8,9,10,11], anthrax [12], chemical warfare-stimulant [13], and glucose level in patients [14]. It has also been explored for environmental protection [15], study of chemical catalysis, [16] and trace of explosive-agents for safety and defense purpose [17]. However, until today, the great potential of SERS biochemical detection has not been fully materialized due to five challenges: (1) it is extremely difficult to make surface-plasmonic-resonant (SPR) nanostructures that can provide a large number of uniform and well-reproducible hotspots for repeatable SERS enhancement; (2) it is even more arduous to robustly obtain ultrahigh sensitivity from the plasmonic structures to detect a broad spectrum of species; (3) most of the state-of-the-art SERS sensing requires the searching effort for hot-spots, which is time-consuming and irrational; (4) it is arduous to translate SERS in *in-vitro* to *in-vivo* applications due to the abundantly available biospecies, which made it difficult to assign Raman signals to specific molecules, as well as the daunting task in characterizing the SERS performance in the *in-vivo* environment. Finally, the SERS detection is still in a static and passive fashion.

In this review, we introduce the fundamental physical principles of surface enhanced Raman scattering, discuss the state-of-the-art progress on innovative fabrication of SERS substrates, and present our recent work on the design, fabrication, characterization, and robotization of Raman nanosensors. Our nanosensors were rationally designed with a longitudinal tri-layer structure that provides well-reproducible high hotspot density of >1200/µm^2^ and an enhancement factor of ~10^10^, and can be motorized by electric and magnetic tweezers for various applications. The motorized SERS nanosensors were applied in predicable molecule location detection, single-cell bioanalysis, and tunable molecule release and detection. This research, exploring the integration of SERS with NEMS, is innovative in design and device concept, which could inspire a new device scheme for various bio-relevant applications. 

### 1.2. SERS Enhancement Mechanisms

As discussed before, the effect of SERS can be generally attributed to two mechanisms: the electromagnetic enhancement and chemical enhancement mechanisms. 

#### 1.2.1. Electromagnetic Enhancement 

**Figure 1:**
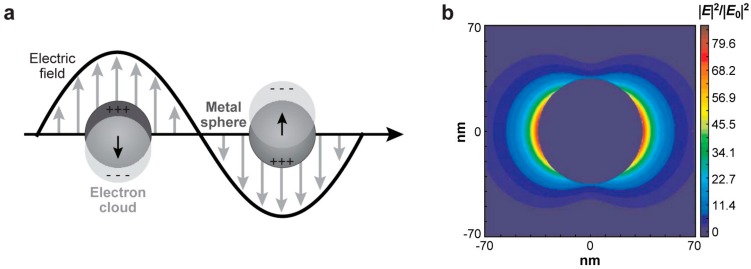
(**a**) Illustration of the localized surface plasmon resonance; (**b**) Extinction coefficient (ratio of cross-section to effective area) of a spherical silver nanoparticle of 35-nm in radius in vacuum. |***E***|^2^ contours for a wavelength corresponding to the plasmon extinction maximum. Peak |***E***|^2^/|***E***_0_|^2^ = 85. Reproduce with permission from [19].

When an electromagnetic wave interacts with metal nanoparticles, the localized surface plasmon occurs, where the conduction-band electrons in a metal nanoparticle collectively oscillate. (Figure 1a) As a result, substantially enhanced *E***-**fields can be found in the vicinity and junctions of the nanoparticles (Figure 1). The locations that have the enhanced *E***-**field are also called hotspots. For molecules in the hotspots, their Raman scattering signals can be dramatically boosted. The enhancement factor of a single molecule (*SMEF*) due to such an effect can be simply expressed as [18]:

(1) $$SMEF\approx M_{Loc}(\omega_{L})M_{Rad}(\omega_{R})\approx \frac{{\left|E_{Loc}(\omega_{L})\right|}^{2}}{{\left|E_{Inc}\right|}^{2}}\frac{{\left|E_{Loc}(\omega_{R})\right|}^{2}}{{\left|E_{Inc}\right|}^{2}}$$

where *M_Loc_* is enhancement of the local field intensity, *M_Rad_* is the radiation enhancement factor, *ω_L_* and *ω_R_* are the resonant angular velocities of the local (*E_loc_*) and radiation field (*E_rad_*), respectively. *E_inc_* is the incidental *E***-**field. In many cases, the Raman shift is small and thus an approximation of *ω_L_* ≈ *ω_R_* can be made. This lead to the widely known expression of the SERS enhancement factor as $SMEF\approx {\left|E_{Loc}(\omega_{L})\right|}^{4}/{\left|E_{Inc}\right|}^{4}$.

Therefore, it can be readily known that enhancement of the localized *E***-**field is the most effective method to increase the SERS sensitivity. For instance, if the plasmonic resonant enhances the localized *E***-**field by 10 times, the Raman signals of molecules can be increased by 10,000 times. Indeed, by optimizing the size, materials, and junction features of plasmonic nanoentities, a SERS enhancement factor (EF) of 10^10^ and above can be achieved [20]. The electromagnetic enhancement is the dominating mechanism for the as-observed ultrahigh EF. 

According to Equation (1), it can be readily seen that both the incident and the Stokes scattered fields should be enhanced to achieve the maximum enhancement factor. It is of great interest to systematically study the optimum excitation wavelength relative to the spectral position of the localized surface plasmon resonance (LSPR) extinction. With the wavelength-scanned surface-enhanced Raman excitation spectroscopy (WS SERES), McFarland *et al*. [21], experimentally proved that the highest SERS EF can be obtained when the wavelength of the excitation source has a higher energy than that of the spectral maximum of the LSPR extinction and is blue shifted to it. For each individual vibrational mode, the maximum enhancement can be obtained when the energy of the excitation laser is in the middle of the Raman shift and the LSPR spectral maximum, where both the incident and Raman scattered photons can be strongly enhanced. Therefore, not all the vibrational modes of molecules can be uniformly enhanced in one SERS test [21,22]. These understandings are important for rational design and optimization of SERS substrates. 

#### 1.2.2. Chemical Enhancement

The electromagnetic enhancement mechanism cannot fully explain the magnitude of SERS enhancement. Evidences showed that there should be a second enhancement mechanism which works independently of the electromagnetic enhancement. For instance, the SERS intensities of CO and N_2_ molecules differ by a factor of 200 at the same experimental conditions [23], while electromagnetic enhancement should not depend on molecular species. 

**Figure 2:**
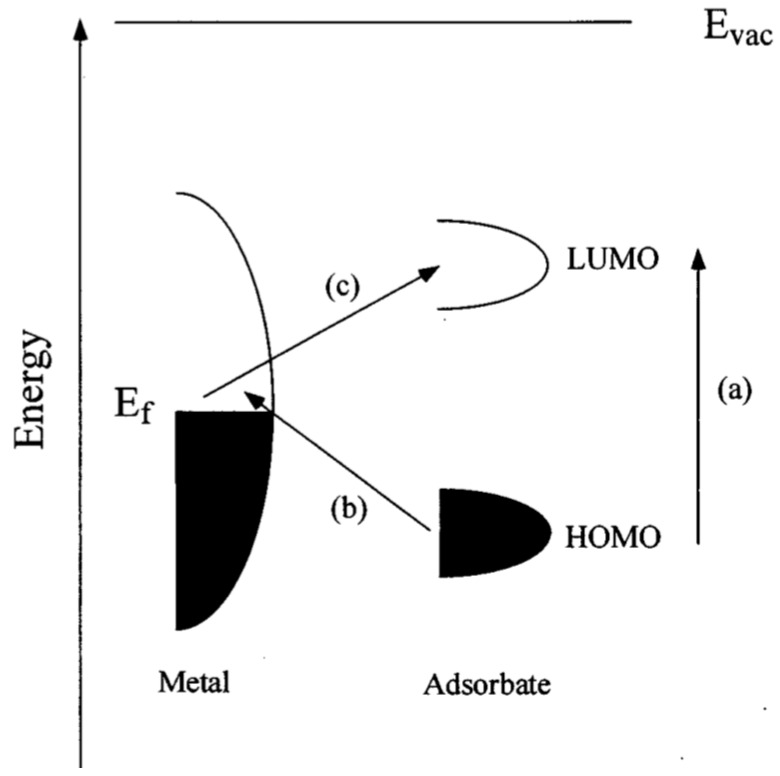
Typical diagram of energy band of a molecule adsorbed on a metal surface. The occupied and unoccupied molecular orbitals are broadened into resonances by their interactions with the metal states; orbital occupancy is determined by the Fermi energy. (a–c) A possible charge transfer excitation is shown. With permission from [23].

These observations can be explained by a resonance Raman mechanism (chemical enhancement) in which the new electronic states arising from chemisorption serve as resonant intermediate states in Raman scattering. The highest occupied molecular orbital (HOMO) and lowest unoccupied molecular orbital (LUMO) of the adsorbate can be symmetrically positioned in the energy band diagram with respect to the Fermi level of the metal (Figure 2). In this case charge-transfer excitation (either from the metal to the molecule or vice versa) can occur at around half of the energy of the intrinsic excitation energy of the adsorbate, which greatly increase the number of excited electrons and thus Raman signal. Research showed that the magnitude of the chemical enhancement is usually 10–100 [23]. 

Researchers worked on the development of a comprehensive theory to describe the SERS enhancement mechanisms and factors for a long time. Recently, Lombardi *et al*. [24] derived a single expression for SERS enhancement based on Herzberg-Teller coupling, which includes contributions from: (1) the surface plasmon resonance in the metal nanoparticle; (2) a charge-transfer resonance involving transfer of electrons between molecules and the conduction band of the metal; and (3) resonances within the molecules themselves. In the study, they demonstrated that the three types of resonances were tightly bonded by Herzberg-Teller vibronic coupling terms and could not be treated individually. However, they did not consider the non-resonance changes in the molecular polarizability that occurs upon adsorbing to the metal surface. Valley and his co-workers [25] studied both normal and surface-enhanced Raman spectra for a set of substituted benzenethiols. They found that the enhancement obtained by experiments varies by a factor of 10 as a result of chemical substitution. Stronger electron donating groups on the benzene unit lead to higher enhancement. The experimental results agree well with the calculation from the static polarizability derivatives determined by the time-dependent density functional theory (TDDFT).

## 2. State-of-the-Art SERS Substrates

**Figure 3:**
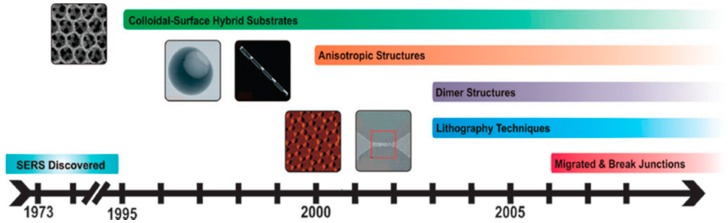
Progress on development of suitable SERS substrates, with permission from [26].

Based on the understanding of the fundamental mechanisms of SERS, intensive interest is focused on fabricating SERS nanostructures with optimal materials, sizes, and configurations for significant enhancement of Raman signals of molecules (Figure 3). 

In general, four typical types of SERS nanostructures emerged with large electromagnetic enhancement: (1) roughened surfaces, e.g., rough Ag surfaces; (2) nanoparticles with controlled aggregations that form nanogaps, e.g. dimers and trimers; (3) nanostructures with sharp tips; and (4) designed core-shell nanostructures [27,28,29,30,31]. As a few thousand papers have been published on the fabrication of SERS substrates in the last decade, this paper will not discuss all approaches used for fabricating SERS substrates but focus on the recent advances according to the most commonly employed synthesis techniques, including wet chemical synthesis, lithography patterning, and bio-assisted fabrication, with elaboration on representative work.

### 2.1. Wet Chemical Synthesis

Wet chemical synthesis methods such as hydrothermal fabrication have been broadly adopted for large-scale efficient growth of monodispersed plasmonic nanoparticles [32,33]. The sizes and shapes of the plasmonic nanocrystals can be precisely controlled by the temperature, concentration and stoichiometry of reagents, as well as surfactants or additives. However, most of the as-synthesized nanoparticles are dispersed as suspensions without controlled aggregations, while nanoparticle pairs with narrow junctions are of dire needs due to the ultra-strong localized surface plasmon resonance (LSPR). In the early days, salt solution was added during the drying process of nanoparticles, which can result in compact aggregation of the plasmonic nanoparticles and narrow junctions between the nanoparticles. Although, SERS EF as high as 10^12^ to 10^14^ has been reported [6,7], the control of the junctions of nanoparticles (hotspots) are adversely random in EF enhancement, quantity, and location [34]. Interestingly, to obtain dimer/trimer/small aggregate structures, transparent silica or ploymers were designed to enclose or link two or more plamonic nanoparticles in individual capsules [35,36,37]. Moreover, assisted with the nanoporous templates, nanopeapods were synthesized and used for intracellular pH sensing [38], which represents a great advance in creating controllable hotspot junctions with colloidal nanoparticles, although the number of nanoparticles in each peapod is random. 

**Figure 4:**
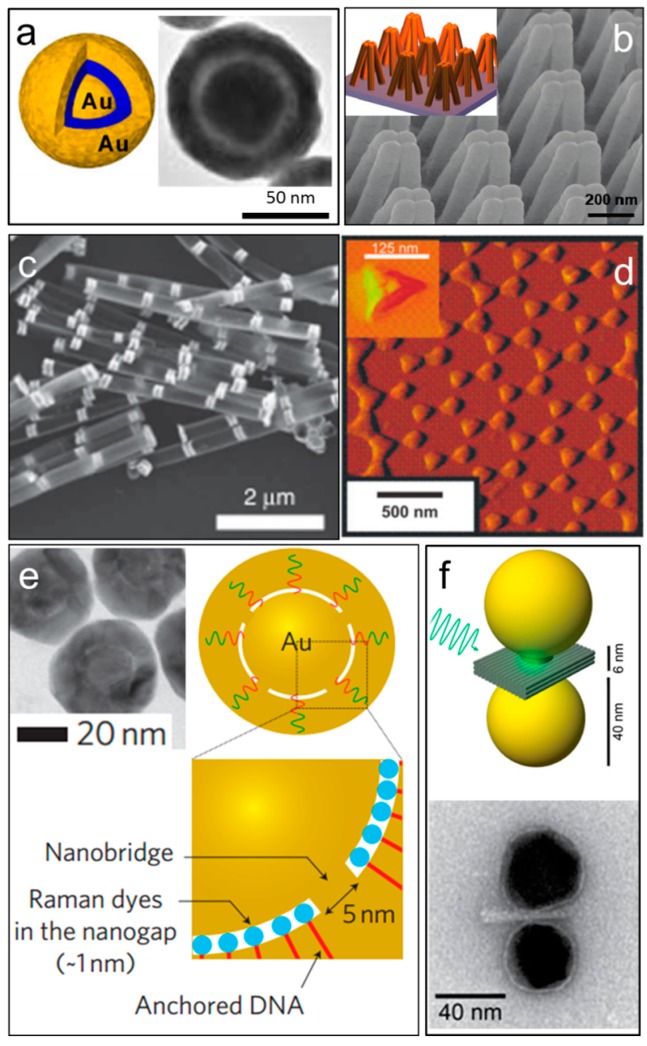
(**a**) Schematic and SEM image of a gold Nanomatryoshka particle, with permission from [41]; (**b**) Schematic and SEM images of gold nanofingers arrays, with permission from [50]; (**c**) On wire lithography: pairs of gold nanodisk with nanogaps, with permission from [51]; (**d**) Nanosphere lithography: tapping-mode AFM image of a representative nanoparticle array. Inset: a close up image of one triangle, with permission from [52]; (**e**) Schematic and SEM images of the gold shell particles template on DNA molecules, with permission from [53]; (**f**) Schematic and SEM images of DNA-origami nanoantennas built from two gold nanoparticles linked via a three-layered DNA origami block at a separation distance of 6 nm, with permission from [54].

In another approach, Au (or Ag) nanoshells received consideration attention. Typically, Au (or Ag) nanoshells were synthesized via deposition of Au seeds (1–2 nm) on monodisperse silica spheres followed by the growth of Au nanoshells to fully cover the entire surface of the silica spheres. Based on this structure, Au/Ag hollow shell assemblies were fabricated and applied as the near infrared SERS probes which can readily detect Raman signals of molecules in 8 mm deep animal tissues [39]. However, nanoparticles in suspension can degrade over time and thus jeopardize their SERS enhancement. It is essential to create plasmonic nanoentities that can be stored in suspension for a long time. Shell-isolated nanoparticles, e.g., Au particles coated with ultrathin silica or alumina shells, that are well protected from molecule contamination and reaction with the solution were synthesized, and thus offer long shelf lifetime [40]. Related to such structures, a large number of core/shell based nanoentities were synthesized, including multilayer Au nanoshells, the so-called nanomatryoshka (Figure 4a) [41]. By tuning the dimensions of cores and shells, the plasmonic resonant frequency of the nanoparticles can be monotonically controlled from the visible to the infrared regime [42,43,44]. Particularly, bimetallic and bi-functional core/shell structures received considerable interest. In such structures, one of the metallic components is SERS active and can be readily applied to monitor the catalytic reactions on the other material component. The bimetallic core/shell structures are fabricated into different shapes, including nanoraspberries [45,46] and starfish [47]. Finally, besides the chemical synthesis as mentioned above, template-assisted [48] and polymer mediated wet-chemical growth [49] were also employed for the fabrication of SERS nanoparticles. 

### 2.2. Top down Lithography Methods

Lithographical techniques, such as photolithography, are broadly used in semiconductor industry. Among all the lithography techniques, the-state-of-the-art electron beam lithography (EBL) can make ultrafine plasmonic nanostructures in an ordered array [55]. However, the high cost of EBL limits the practical applications of the devices. In addition to EBL, Gartia *et al.*, demonstrated that freestanding nanopillars coated with silver can be fabricated by laser interference lithography on a six inch wafer and applied such structures for effective SERS detection. The SERS substrate offers an ultrahigh-uniformity and an average homogeneous enhancement factor close to 10^8^ [56]. With such kind of SERS substrates, even volatile compounds, such as toluene vapor, can be detected. A simple adsorption model was also developed from the temporal evolution of SERS signals.[57] In parallel, the nanoimprint lithography, developed by Chou *et al.* [58], emerged as an economic alternative technique of EBL for mass production of nanostructures with high precision. A notable work is reported by Hu *et al.*, who successfully created ordered arrays of gold-capped-polymer nanofingers in a large scale by nanoimprint lithography [50,59], where designed numbers of nanofingers can be snapped together on the finger tips and form narrow junctions due to the surface tension generated in the process of solvent evaporation (Figure 4b). The EF in the junctions is estimated as ~10^10^, one of the highest among the state-of-the-art. [50,59] Similar to the concept of snapped fingers, Schmidt *et al.*, explored another route to economically create hotspots on a wafer scale in assembled silver-capped Si nanopillars via maskless reactive ion etching [60]. By assuming that only a few molecules are trapped in the hot spots, which account for the detected Raman signals, an EF of ~10^11^ is estimated. Recently, silver nanoparticle islands over a silver mirror with a SiO_2_ spacer layer was fabricated by the standard sputtering and evaporation techniques. Such SERS substrates can detect an unprecedented number of single molecule events (>7000) [61]. 

### 2.3. Reductive Fabrication Based on Selective Etching

In addition to the aforementioned approaches, unique etching methods, such as on-wire lithography [51,62,63,64] were developed to make SERS substrates with junctions of a few nanometers for ultrahigh and well reproducible enhancement (Figure 4c). The on-wire lithography technique relies on fabricating designed multi-segment nanowires with segments made of distinct chemistry before removing selected segments to create gap structures. Note that the nanowires are conformably coated with a silica film to fix the relative positions of the metal segments and thus the gap sizes before the selective etching. As a result, arrays of a few nanometer junctions can be rationally created between the unetched nanodisk/rod pairs (Figure 4c), which demonstrate single-molecule sensitivity in detecting various biochemicals such as methylene blue [64], *p*-mercaptoaniline [65], and Cy-3-labeled DNA [66]. However, the number of hotspots that can be created on each nanowire is limited to a few. Also the positions of the nanowire gaps are largely random on a substrate. Therefore it takes great efforts to find hotspots before detecting molecules. The earlier work including nanosphere lithography [52], (Figure 4d) porous template-assisted deposition [67,68], and nanosphere templated nanocrescent fabrication [69] were also explored to create controlled hotspots in a large array for sensitive SERS detection. 

### 2.4. Bio-Assisted Fabrication

Recently, the state-of-the-art DNA-origami [53,54,70] assisted assembling of plasmonic nanoparticles emerges for fabrication of plasmonic nanoparticle pairs with precise geometry, gap size and number of particles. For instance, Lim *et al*. [53] successfully prepared DNA-tailored nanoparticles with 1-nm junctions for highly uniform and reproducible SERS as shown in Figure 4e; Kühler *et al*. [54] innovatively coupled plasmonics with DNA-origami and obtained nanoantennas from two gold nanoparticles linked by a three-layered DNA origami block with a separation distance of 6 nm (Figure 4f).

## 3. Robotic SERS Sensors

Despite all the aforementioned progress in the fabrication of SERS substrates, the practical applications of SERS for biochemical detection remain a grand challenge because: (1) it is difficult to create a large number of uniform hotspots for well-repeatable SERS detection at a low cost. We note that a major effort in all of the previously discussed fabrication explorations is to obtain reproducible SERS substrate with controlled sizes of hotspots. This task is extremely challenging. According to Equation (1), the enhancement of Raman signals increases with *E*^4^ and the strongest SERS are largely obtained from nanojuntions of a few nanometers in sizes. As a result, variation of the junctions of a few nanometers can result in SERS fluctuation as high as an order of magnitude. This presents a grand challenge to obtain reproducible and uniform SERS substrate with current fabrication techniques; (2) it remains arduous to obtain ultra-sensitivity from the hotspots for detection of a broad spectrum of species due to the difficulties in controlled the sizes of hotspots to as small as a few nanometers; (3) It is even more challenging to realize location predicable sensing for rapid detection without the time-consuming searching effort; (4) it remains a grand challenge to translate the in-vitro applications of SERS for in-vivo study due to the abundantly available biospecies, which made it difficult to assign Raman signals to specific molecules, as well as the daunting task in characterizing the SERS performance in the *in-vivo* environment. Finally, most of the state-of-the-art sensors detect biospecies in a passive and static fashion.

### 3.1. Design of the Nanosensors

**Figure 5:**
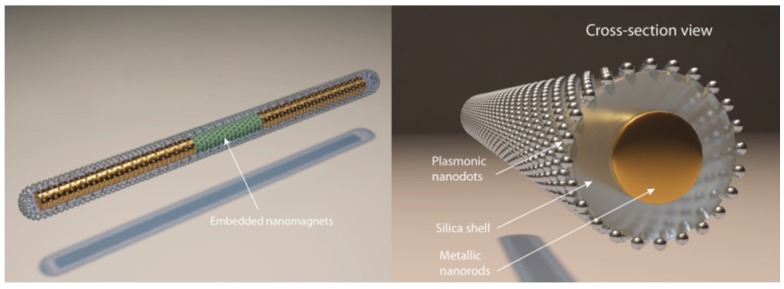
Structure of a tri-layer nanocapsule [71]. With permission from [71].

In our recent research, we explored to resolve the aforementioned problems by rational design, fabrication and robotization of a unique type of nanocapsule SERS sensors (Figure 5). 

The nanocapsule sensor has a tri-layer longitudinal structure with a three-segment Ag/Ni/Ag nanorod serving as the core, a thin layer of silica in the center, and uniformly distributed Ag nanoparticles at the outer layer. The inner metallic nanorod core is critical for realizing the robotization of nanosensors, which can be electrically polarized and manipulated efficiently by the electric tweezers—a recently developed nanomanipulation technique based on the combined AC and DC *E***-**fields [72]. The presence of the Ni segment with controlled aspect ratio in the metallic nanowire also allows versatile magnetic manipulation and assembling [73,74]. Thus the plasmonic nanocapsules can be manipulated by either electric or magnetic tweezers [75,76]. The central silica layer provides a supporting substrate for the synthesis of Ag nanoparticles and also serves to separate the Ag nanoparticles from the metallic nanorod core to avoid plasmonic quenching. Finally, a uniform layer of Ag nanoparticles with optimized sizes and junctions is grown on the surface of silica, which provides a large number of hot spots (~1200/µm^2^) and an enhancement factor (EF) of ~1.1 × 10^10^ for ultrasensitive SERS detection.

### 3.2. Fabrication Procedure

**Figure 6:**
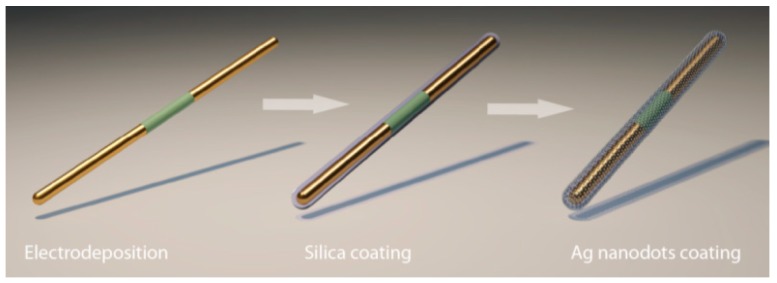
A scheme of the synthesis process of plasmonic magnetic nanocapsules, with permission from [71].

The process for fabrication of the nanocapsules is shown in Figure 6. In the first step, Ag/Ni/Ag nanorods (300 nm in diameter) were synthesized by electrodepositing Ag and Ni from their respective electrolytes into nanoporous anodized aluminum oxide (AAO) membranes. In brief, a three-electrode electrochemical setup was configured with the copper layer at the back of AAO membranes serving as the working electrode, Ag/AgCl as the reference electrode, and Pt as the counter electrode. The materials of Ag or Ni were electrodeposited at −1 V (*vs*. Ag/AgCl reference electrode) from their respective elecrolytes into the nanopores. The amount of electric charges passing through the circuit controlled the length of each segment of the nanowires; and the size of the nanopores determined the diameters of the nanowires. After selective etching the copper and AAO membrane, the nanowires were released in suspension, sonicated, and centrifuged in deionized (D.I.) water and ethanol, alternatively, before resuspended in D.I. water [63]. 

In the second step, silica was uniformly coated on the outer surface of the Au/Ni/Au nanowires via hydrolysis of tetraethyl orthosilicate [71]. The Ag-Ni-Ag nanowire suspension of 0.4 mL (5.7 × 10^8^ counts/mL, in D.I. water) was mixed with TEOS (0.8 mL), ammonia (0.2 mL), ethanol (3 mL), and D.I. water (1.6 mL). Then the mixture was sonicated for 2 h and the silica coated nanowires can be collected.

**Figure 7:**
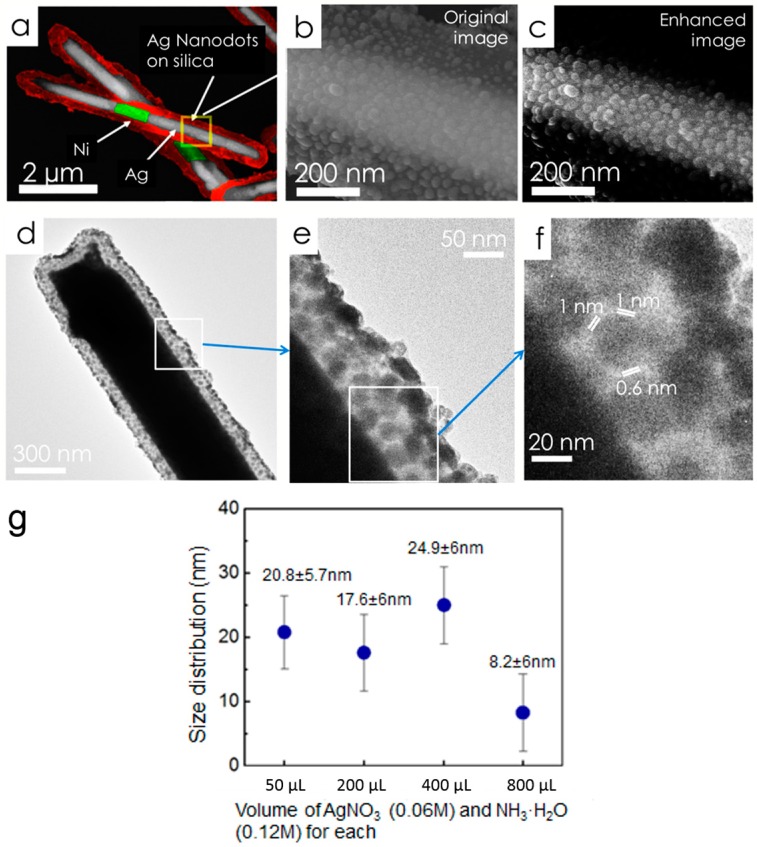
Color enhanced SEM images of tri-layer nanocapsules at (**a**) low magnification and (**b**) high magnification; (**c**) The contrast enhanced image of (**b**); (**d**,**e**) TEM images of a typical nanocapsule show a fairly uniform distribution of Ag NPs; (**f**) Arrays of junctions of the Ag NPs <2 nm; (**g**) By controlling the amount of the reactants of silver nitrate and ammonia dispersed in in Ag/Ni/Ag nanorod suspension (5.7 × 10^8^/mL, 400 µL), the average particle size of Ag NPs can be tuned from 8 nm–25 nm. With permission from [71].

In the last step, Ag nanoparticles (NPs) were grown on the silica surface of the nanowires. In brief, freshly prepared silver nitrate (AgNO_3_, 0.06 M, 400 µL), ammonia (NH_3_·H_2_O, 400 µL, 0.12 M), and silica coated Ag/Ni/Ag nanorods (5.7 × 10^8^/mL, 400 µL) were mixed and stirred for 1 h to ensure the adequate adsorption of Ag(NH_3_)_2_^2+^ ions on silica. Next, polyvinylpyrrolidone (PVP) in ethanol (10 mL, 2.5 × 10^−5^ M) was added to the mixed solution and the temperature was kept at 70 °C for 7 h before washed with ethanol and acetone for several times to remove the surface residue for characterization. As a result, a large number of nanocapsules with uniform Ag NPs distributed on the silica surface of Ag/Ni/Ag nanowires can be obtained as shown in Figure 7a,b. By controlling the amount of the reactants of silver nitrate and ammonia in Ag/Ni/Ag nanorod suspension (5.7 × 10^8^/mL, 400 µL for each), the average particle size of Ag NPs can be tuned from 8 nm–25 nm as shown in Figure 7g. The highest Raman EF of 1.1 × 10^10^ was obtained from nanocapsules as shown in the enhanced scanning electron microscopy (SEM) image in Figure 7c [77]. The SEM characterization showed that the Ag nanoparticles have an average size of 25 ± 6 nm and density of ~1600/µm^2^. The transmission electron microscopy (TEM) images in Figures 7d–f show a high density of narrow junctions uniformly distributed on the nanocapsules. It is known that the smaller the junction sizes between neighboring Ag NPs, the stronger the enhancement of the *E***-**field due to the plasmonic resonance. If we only consider those junctions of <2 nm as hotspots, where the major Raman signals collected from molecules come from, the hotspot density can be estimated at 1200/µm^2^. 

### 3.3. SERS Characterization

**Figure 8:**
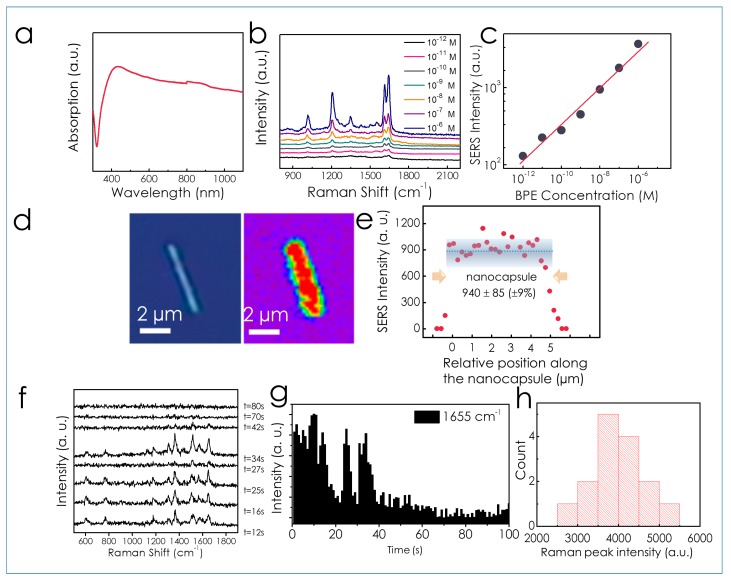
(**a**) Optical absorption spectrum of the nanocapsules without metal cores; (**b**) SERS spectra of BPE from 1 pM to 1 µM; (**c**) The SERS intensity increases with concentrations of BPE at 1644 cm^−1^; (**d**) SERS mapping profile of 1 µM R6G dispersed on a nanocapsule shows uniform SERS intensity on the surface of the nanocapsules (**e**) SERS intensity distribution along a single nanocapsule; (**f**) On/off SERS spectra of R6G molecules in a 100-sec time frame with 1-sec integration for each spectrum; (**g**) SERS Intensity fluctuation as a function of time (@1655 cm^−1^) (with permission from [71]); (**h**) SERS intensity of 100 nM nile blue solution @ 595 cm^−1^ detected from different nanocapsules fabricated in the same batch.

The optical absorption of the nanocapsules was studied to find the optimal condition for SERS detection. The Ag/Ni/Ag metal-cores were removed from the nanocapsules to avoid the effect on the absorption measurement. The nanocapsules exhibit an absorption peak close to 450 nm due to the collective plasmonic resonance of assembled Ag nanoparticles (Figure 8a) [78,79]. The absorption peak showed a broad background, which can be attributed to the distribution of the size and shapes of the Ag nanoparticles. A 532 nm laser was chosen as the excitation source for the SERS characterization, which matches fairly well with the absorption of the Ag nanoparticles.

To characterize SERS performance of the as-obtained nanocapsules, we employed the commonly used molecules 1,2-bi-(4-pyridyl) ethylene (BPE) and rhodamine 6G (R-6G) as the analytes. To ensure the repeatability of the experiments, we sparsely dispersed the nanocapsules in a 3 mm-diameter well made of a 1 mm-thick polydimethylsiloxane (PDMS) film. BPE molecules (10 µL in ethanol) with concentrations from 1 pM (10^−12^ M) to 1 µM (10^−6^ M) were dispersed into the PDMS well, sealed with a glass cover slip, and incubated for 10 min. Then the nanocapsules were carefully rinsed three times with ethanol to remove the excess BPE molecules on the surfaces. SERS spectra were collected from a single focusing spot (~1 μm) with an integration time of 5 s by using a confocal Raman microscope as shown in Figure 8b. The intensity of SERS increases with the molecule concentration monotonically (Figure 8c).

We estimated the SERS EF of the nanocapsules detected from the non-resonant BPE molecules following a widely used method [50,60] given by $EF=\frac{I_{SERS}/N_{SERS}}{I_{RS}/N_{RS}}$, where *N_SERS_* is the average number of adsorbed molecules enhanced by the SERS substrate. *I_SERS_* is the corresponding SERS intensity, *N_RS_* is the average number of molecules excited without surface enhancement, and *I_RS_* is its corresponding SERS intensity. The values of *IRS* were obtained by detecting SERS intensity of 0.1 M BPE in ethanol enclosed in a polydimethylsiloxane (PDMS) well of ~5 mm in diameter with a 532 nm laser through a 50× objective. The size of the laser spot is ~1 µm. The spectra are collected by a Acton SpectraPro spectrograph (Princeton Instrument, Trenton, NJ, USA) coupled ProEM ultrasensitive CCD camera (Princeton Instrument, Trenton, NJ, USA). The total number of molecules without SERS enhancement (*N_RS_*) is estimated from the dimensions of the laser spot and the effective detection volume height using a method reported previously [80]. To determine the number of molecules that contribute to the SERS signals, we assumed that only molecules residing in the 1.6 nm^3^ volume of the 1.17 ±0.5 nm narrow junctions contributed to the measured Raman intensity. The estimated EF is 1.1 × 10^10^, which is among the highest of the state-of-the-art SERS sensors. The detailed calculation is given in the supporting information of the reference [71]. The distribution of the EF can be suggested by the SERS intensity detected from different nanocapsules fabricated in the same batch (100 nM Nile Blue solution) (Figure 8h). The results show a maximum variation of around 1.7 times among 15 test nanorods, suggesting the average EF could also have a distribution among different of nanocapsules of the same magnitude of ~2 times.

Note that the method for evaluating SERS enhancement factor can be subjective in the research community. It depends on whether all the molecules in the detection region or just those located at the hot spots are countered towards the calculation [60,81]. We also calculated the EF based on the entire area of the nanorods excited by the laser beam. It showed a value of 1.8 × 10^7^. This estimation is more like an averaged estimation, which is still a high EF without the consideration that most SERS signals are from the hotspot at the junctions of Ag nanoparticles. In fact, on the nanoscale, the EF of SERS changes drastically with locations. It depends on whether it is on the hotspot between the junctions or just on the surfaces of Ag nanoparticles. It also depends on the sizes of the Ag nanoparticles and junctions, so the overall estimation of EF in the range of 1.8 × 10^7^ to 1.1 × 10^10^ should give a good evaluation of the SERS performance of the nanocapsules. 

Moreover, we detected time-dependent Raman spectra of molecules from the nanocapsules. R6G molecules with a concentration of 1 pM were functionalized on the nanocapsules using the aforementioned method. The Raman signals were collected for 100 s with each spectrum taken for 1 s. They fluctuated and even disappeared sometimes as shown in Figure 8f–g, which is consistent with single-molecule behaviors that molecules diffuse in and out of hotspots. Similar behaviors are observed on different substrates, such as gold-silver core-shell nanodumbbells and cluster of silver colloids [6,34,82,83]. We note that according to the bi-analyte study on silver colloids [20,83], an EF on the order of 10^7^–10^8^ is sufficient for detecting single molecules of various species, such as R6G, 1,2-di-(4-pyridyl)-ethylene [20]. The estimated EF of our substrate is as high as 10^10^. This suggests it possible that the sensitivity of the nanocapsules is in the single-molecule regime. While, we note that the above study is not the absolute evidence. More rigid experiments, such as bi-analyte tests, should be conducted for confirmation [84]. 

Besides the ultrahigh sensitivity, the SERS signals of molecules dispersed on the nanocapsule surfaces are largely uniform on areas of 1 µm diameter, where the SERS spectra of R6G (1655 cm^−1^, 1 µM) are collected by scanning a ~1 µm-diameter laser spot along the length of the nanocapsule. The step size is 250 nm and the integration time for each spectrum is 0.5 s. It shows that the SERS enhancement was largely uniform on the surface of a nanocapsule and gradually reduced to zero on the edges due to the curved structures (Figure 8d). Analysis shows that the SERS intensity varied by only ±9% along the nanocapsules in Figure 8e, which is a very narrow distribution in SERS detection. This uniformity can be attributed to two factors: (1) the largely uniform size and distribution of the Ag nanoparticles give relatively low intensity variation of hotspots; (2) the average effect due to the high density of hotspots in each detection area (1200 hotspots/μm^2^, ~240 in each detection area). As a result, the SERS intensity shows good uniformity along the nanocapsules due to the averaged EF from all the hotspots. It also suggests that the nanocapsules can detect monolayer analytes with reasonable repeatability. 

### 3.4. Further Enhancement of SERS

**Figure 9:**
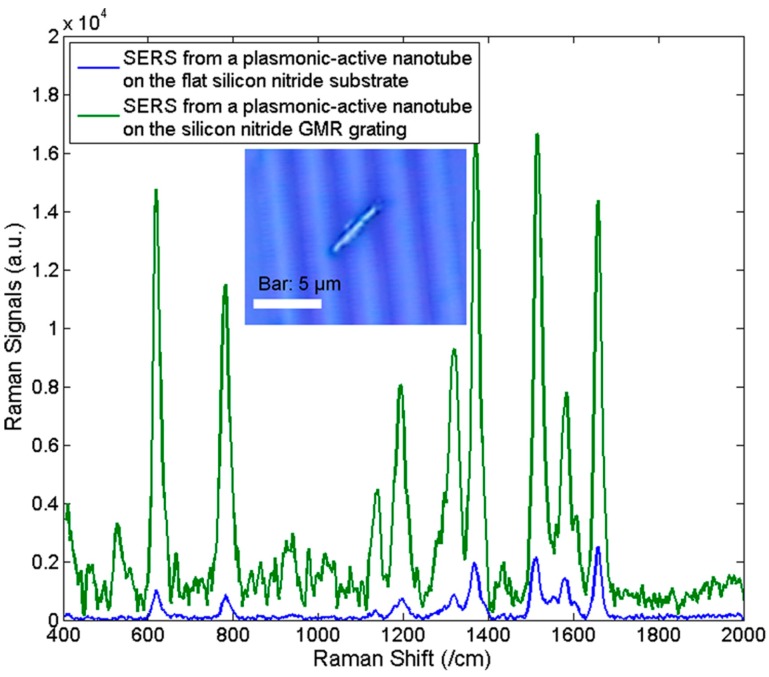
Raman Spectra of 1 µM R6G detected from a plasmonic-active nanotube on a flat Si_3_N_4_ substrate (blue) and from a plasmonic-active nanotube on the GMR optical grating (green). With permission from [85].

We further improved Raman enhancement by ~10 times with optical gratings placed underneath the nanocapsules by collaboration with Dr. Wang in Oregon State University and others [85]. The enhancement is due to the coupling between the guided mode resonance (GMR) [86,87,88] and strong localized surface plasmon polaritons (LSPPs). If we just randomly disperse a large number of metallic nanoparticles on top of dielectric gratings, the GMR resonant mode of the optical grating can be greatly damped and the SERS enhancement becomes weakened [85]. Therefore, metal nanoparticles have to be lithographically patterned at suitable spots on the photonic grating [86] to reduce the problems of weakened resonance. The use of the nanocapsules can controllably deliver a limited number of silver nanoparticles to closely couple with the grating in a non-lithographical manner, which could minimize the metal absorption to the GMR mode while still providing sufficient “hot spots” for SERS sensing. From the nanocapsules coupled on optical gratings, Raman signals of R6G molecules can be increased by ~8–10 times across the entire Raman spectrum compared to those without gratings (Figure 9). As a result, the EF can be further improved to ~10^11^ by combining nanocapsules with GMR gratings.

**Figure 10:**
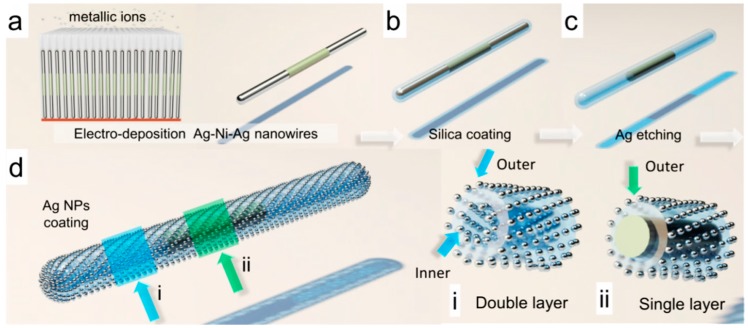
Schematics of the fabrication process of the partially hollow nanotubes. (**a**) Electrodeposition of Ag/Ni/Ag nanowires; (**b**) Silica shells coating; (**c**) Etching of Ag segment to get hollow nanotubes with solid Ni embedment; (**d**) Synthesis of Ag NPs on both the inner and outer surfaces of nanotubes. (Inserts are cross-section view of (i) the hollow segment and (ii) the Ni embedded segment of the nanotubes.) (With permission from [73]).

**Figure 11:**
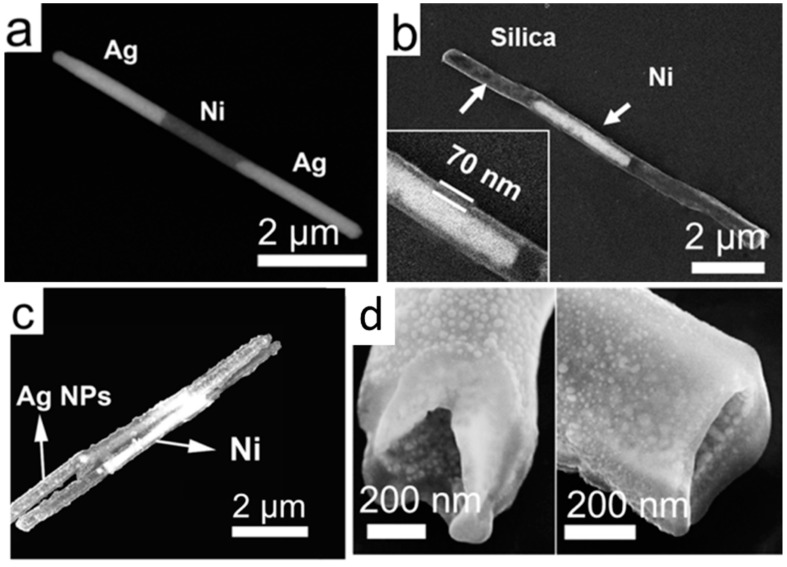
Scanning Electron Microscopy images of (**a**) multisegment Ag/Ni/Ag nanowires; (**b**) silica nanotubes embedded with Ni nanomagnets; (**c**) silica nanotubes with Ni segments and surface-coated Ag NPs; (**d**) cross-sectional images of nanotubes obtained by FIB milling show the nanotubes are hollow with Ag NPs on both the inner and outer surfaces. With permission from [73].

**Figure 12:**
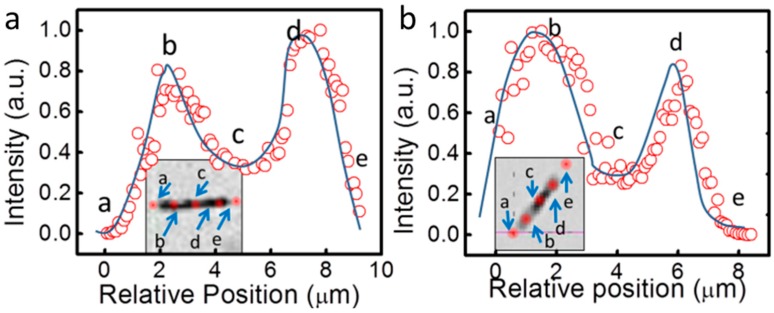
(**a**) Ni embedded and (**b**) Pt embedded hollow nanotubes. With permission from [73].

In addition to the enhancement from nanophotonic devices, the SERS EF of the nanocapsules can also be increased by the near-field effect. We designed a partial hollow nanotube structure, where Ag nanoparticles were grown on both the inner and outer surfaces of the silica nanotube as shown in Figure 10 and Figure 11. The fabrication of such nanotube structures was achieved by re-designing the synthetic process of the aforediscussed three-layer nanocapsules, where after coated by silica layers of 70 nm in thickness, the Ag segments of the Ag/Ni/Ag nanowires (3 µm/3 µm/3 µm) were selectively etched in silica nanotubes with embedded Ni segments before the synthesis of Ag nanoparticles (Figure 10c and Figure 11b). Since the silica layer is porous and solution permeable, the Ag nanoparticles can be readily grown on both the inner and outer surfaces of the hollow segments of the Ni embedded nanotubes as shown in the SEM images of focused ion beam (FIB) processed samples (Figure 10d and Figure 11c,d). The as-grown Ag NPs were semi-spherical and densely arranged, while rarely overlapped. The SERS characterization showed that over 2-fold SERS enhancement can be achieved in the hollow segments compared to that of the solid segments of the nanotubes (Figure 12a,b). 

To understand how the dual-side coating of Ag nanoparticles can further enhance SERS, numerical simulations were conducted with Comsol 3.5a RF by collaboration with Wang [73]. The result suggests that the enhancement of SERS by the dual-side-Ag-coated hollow nanotubes can be attributed to the near-field coupling between the inner and outer layers of Ag nanoparticles (Figure 4 in ref [73]) through the 70 nm thin silica as well as the additional hotspots available on the inner surfaces of the nanotubes. 

## 4. Motorization of SERS Nanosensors and Applications

Although various innovative approaches were explored to make ultrasensitive SERS nanoparticles, to the best of our knowledge, the SERS nanoparticles are either randomly dispersed in suspension [89], on substrates [6], or patterned into arrays [52,90,91,92,93]. In many cases, great efforts have to be spent on searching the hotspots before detection, which is extremely inefficient. Also, the operation of the state-of-the-art SERS sensors remains in a static and passive fashion in large. It is highly desirable to motorize the SERS nanosensors and change the detection scheme into a robotic fashion, which could open up many unprecedented opportunities for biological research. 

### 4.1. Plasmonic Tweezers for the Manipulation of Plasmonic Nanoparticles

**Figure 13:**
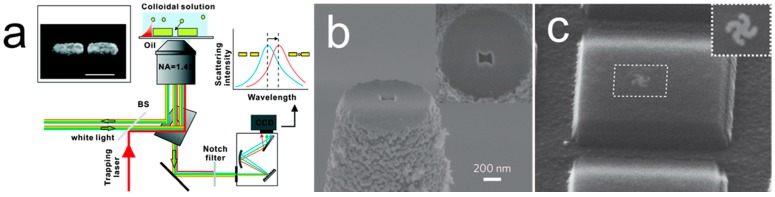
Plasmonic tweezers for trapping and rotating nanoentities. (**a**) Schematic diagram of the setup of the plasmonic tweezers trapping and detecting 10 nm Au particles [94]; With permission from [94]; (**b**) SEM image of an 85-nm-gap bowtie nano-aperture patterned at the extremity of a tapered optical fiber [95]; With permission from [95]; (**c**) SEM image of a light-driven plasmonic motor [96]. With permission from [96].

Plasmonic nano-optical tweezers which utilize the plasmonic effect to capture and manipulate the nanoparticles can be a promising tool for robotization of SERS sensors. The plasmonic effect is especially advantageous in focusing light to a subwavelength region to circumvent the optical diffraction limit and offer an ultrahigh optical intensity. By designing suitable metal nanopatterns, such as pairs of nanoantennas or bow ties, the plasmonic tweezers can manipulate and capture objects as small as tens of nanometers, such as Au nanoparticles, which can be potentially used for SERS detection. As shown in Figure 13a, a pair of Au nanoantennas with a junction of 10 nm can both trap and sense 10 nm Au particles [94]. Based on the same plasmonic effect, self-induced back-action (SIBA) trapping probes, where a nano-aperture in a metallic thin film is lithographed on the tip of an optical fiber, were developed by Quidant, *et al.*, as shown in Figure 13b [95]. The probe can stably trap nanoparticle with a power of at least an order of magnitude lower than those without plasmonic enhancement due to the nano-apertures. 

Moreover, plasmonic nanomotors made of gammadion gold nanostructures have been reported recently, which can rotate clockwise and counterclockwise depending on the frequency of the exciting laser (Figure 13c) [96]. In such a device, both the geometry and the material of the rotor, *i.e.*, gammadion and gold, are designed with optimized plasmonic resonance, which consequently, greatly enhances light-induced mechanical torques. The motors, as small as 200 nm in diameter, can generate sufficient torques to compel the rotation of a surrounding silica microdisk with much larger dimensions (2.2 × 2.2 × 0.3 μm^3^). The nanoparticles manipulated by the plasmonic tweezers have great potentially for applications in SERS detection, although not sufficiently demonstrated. 

### 4.2. Electric Tweezers for the Manipulation and SERS Applications

The unique aforediscussed nanocapsules with a high density of hotspots of ~1600 /µm^2^ and magnetic nanorod embedment can be readily motorized by electric tweezers [71]. They can be transported in both the X and Y directions, rotated with controlled speed and orientation, and demonstrated for applications including biochemical delivery and time-dependent monitoring, single-cell bioanalysis, and position deterministic sensing. 

The electric tweezers is our recently developed nanomanipulation technique based on the combined DC and AC *E***-**fields, which can transport longitudinal nanoentities along desired orientation in aqueous suspension with a precision of 300 nm. The fundamental principles have been reported previously [72,97,98,99,100,101]. In brief, in combined DC and AC *E* fields, a longitudinal nanoparticle can be transported by the DC *E* field due to the electrophoretic force and aligned in the direction of the AC *E* field due to the dielectrophoretic force. The transport and alignment are controlled independently by the DC and AC *E* fields, respectively. As a result, longitudinal nanoparticles such as nanowires can be readily transported along arbitrary trajectories and positioned at designated locations by applying *E* fields in both the X and Y directions with controlled durations. The transport velocity of nanowires linearly increases with the DC *E* field and reaches a speed of at least ~80 µm [71].

#### 4.2.1. Location Predictable Sensing

Using the electric-tweezers, we assembled ordered arrays of nanocapsule SERS sensors on patterned nanomagnets by transporting and fixing the positions of nanocapsules atop of ordered arrays of prepatterned nanomagnets. This effort aims to reduce the labor-intensive efforts in the search of the hotspots before SERS detection. 

**Figure 14:**
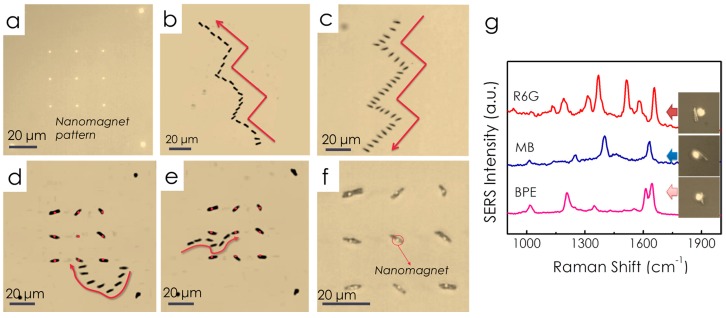
Nanocapsules can be precisely transported and assembled on the pre-patterned nanomagnets with electric tweezers. (**a**) A 3 × 3 nanomagnet array was fabricated by electron beam lithogaphy. With combined AC and DC *E* fields applied in both the X and Y directions, a nanocapsule was moved along prescribed trajectories such as “stairs”, aligning (**b**) parallel and (**c**) perpendicular to its orientation; (**d**,**e**) Overlapped snapshots show the delivering and assembling process of a nanocapsule onto a nanomagnet. Note the nanomagnets were highlighted in red; (**f**) An assembled 3 × 3 nanocapsules array. All the images were taken by reflective optical microscope; (**g**) From the assembled nanocapsules, various chemicals including R6G, methylene blue, and BPE have been detected. With permission from [71].

Uniform AC and DC *E* fields were established in a quadruple microelectrode with the parallel electrode pairs separated at a distance of 500 µm. At the center of the quadruple microelectrode, arrays of nanomagnets were patterned by the electron beam lithography (Figure 14a). Each nanomagnet has a diameter of 1 µm and consists of a trilayer structure with a 6 nm Cr adhesion layer on the glass substrate, 100 nm Ni layer providing magnetic fields, and 100 nm Au layer for tuning the magnetic force. Nanocapsules in D.I. water were dispersed in the center of the quadruple microelectrodes and were transported by applying a combined AC (15 V, 20 MHz) and DC voltages (−2.5 V to +2.5 V) on the quadruple electrodes. By programming the AC and DC *E* fields in both the X and Y direction, we compelled the nanosensors to move along a prescribed trajectory, such as “stairs”, with orientations either parallel (Figure 14b) or perpendicular (Figure 14c to their transport directions. When nanosensors were maneuvered into the vicinity of nanomagnets by the electric tweezers, the magnetic attraction force, between the Ni segments in the core of the nanocapsules and the Ni layer of nanomagnets, secured the nanocapsules on the top of the nanomagnets. The manipulation of the nanocapsules was so versatile and precise that we can easily maneuver a nanocapsule to pass by a few neighboring nanomagnets and anchor it on a nanomagnet in the center of the array (Figure 14d,e). In this manner, we have assembled a 3 × 3 array of nanosensors on top of nanomagnets as shown in Figure 14f, where the bright dots are the optical images of the nanomagnets. Finally, from the assembled nanocapsule arrays, we successfully detected Raman signals of various chemical species including R6G, methyl blue (MB), and BPE and realized location predictable sensing as shown in Figure 14g. 

#### 4.2.2. Live Cell Sensing

**Figure 15:**
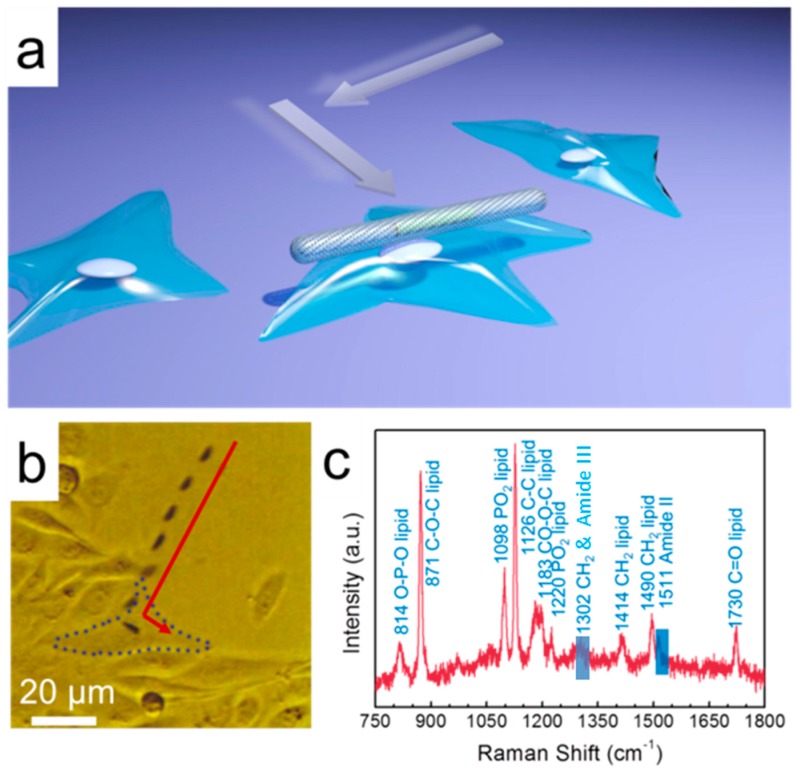
(**a**,**b**) Transport a partial-hollow nanocapsule motor to a single live Chinese hamster ovary (CHO) cell amidst many; (**c**) SERS spectrum shows the CHO cell membrane is primarily lipid. Two peaks highlighted in blue can be attributed to Amide II and Amide III from proteins (blue bars). With permission from [73].

The nanocapsules can also be applied to analyze the membrane composition of single live cells amidst many. Although single complex biological samples can be investigated with standard Raman microscopy, a detailed investigation of specific sub-components on the cell membranes has not been realized with this technique [102]. Here, by leveraging the ferromagnetic property of the nanocapules, we compelled the nanocapsules to a specific living Chinese hamster ovary (CHO) cell amidst many and detect the membrane chemistry with SERS spectroscopy (overlapped images in Figure 15a,b). The SERS spectra were collected from the area of the cell membrane in contact with the nanocapsule with an integration time of 5 s under illumination of a 532 nm laser. The SERS characteristic peaks can be assigned to both proteins and lipids. As shown in Figure 15c, peaks at 814, 871, 1098, 1126, 1183, 1220, 1302, 1414, 1490, and 1730 cm^−1^ can be assigned to lipid tentatively by comparing with results from previous work [89,102,103]. The peak at 1511 cm^−1^ and ~1300 cm^−1^ can be attributed to amide II [102] and amide III [104] from protein molecules, respectively. Note that the ~1300 cm^−1^ band can be assigned to both lipid and amide III. These results revealed that the cell membrane in contact with the nanocapsule motor consists of mostly lipids and some protein molecules, which agrees with our understanding of the chemistry of cell membranes [102]. In the absence nanocapsules, at the same conditions with excitation wavelength of 532 nm and an integration time of 5 s, no intrinsic Raman spectra from the cell can be detected. This clearly demonstrates the highly desirable features of the nanocapsules in manipulation and ultrasensitive detection in single-cell bio-analysis. 

#### 4.2.3. Tunable Release of Single and Multiplex Molecules

**Figure 16:**
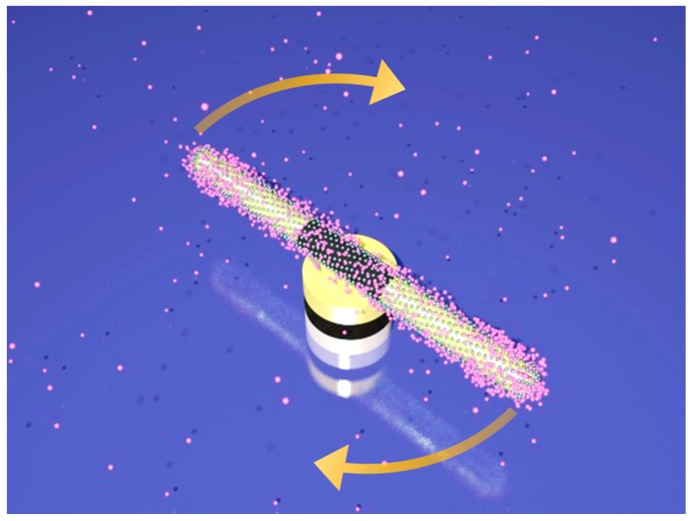
Schematic diagram of molecule release from a rotary plasmonic nanomotor [105]. With permission from [105].

Finally, the nanocapsules were assembled into rotary nanomotors. They were compelled to rotate with controlled angle, speed, and chirality for tunable molecule release and detection. The nanomotors consist of the nanocapsules as rotors, patterned nanomagnets as bearings, and the quadruple microelectrodes as stators (Figure 16) [105]. The electric tweezers were employed to assemble and actuate the rotary nanomotors. The assembling of the nanomotors were achieved by using the same technique employed for assembling nanocapsules into ordered arrays (Figure 14), where the nanocapsules were transported to anchor atop of the nanomagnets. 

By creating rotating *E***-**fields via four AC voltages with sequential 90° phase shifts applied on the quadruple microelectrodes, the nanorod rotors can be compelled to rotate due to the interaction between the polarized nanoparticle and the external *E***-**fields. The electric torque (*T_e_*), proportional to *E*^2^, is countered by the viscous torque (*T_ɳ_*), which is proportional to the rotation speed of the nanoparticles (Ω). As a result, the rotation speed of the nanomotors (Ω) can be precisely controlled by the applied *E***-**fields with a linear dependence to *V*^2^ [106].

**Figure 17:**
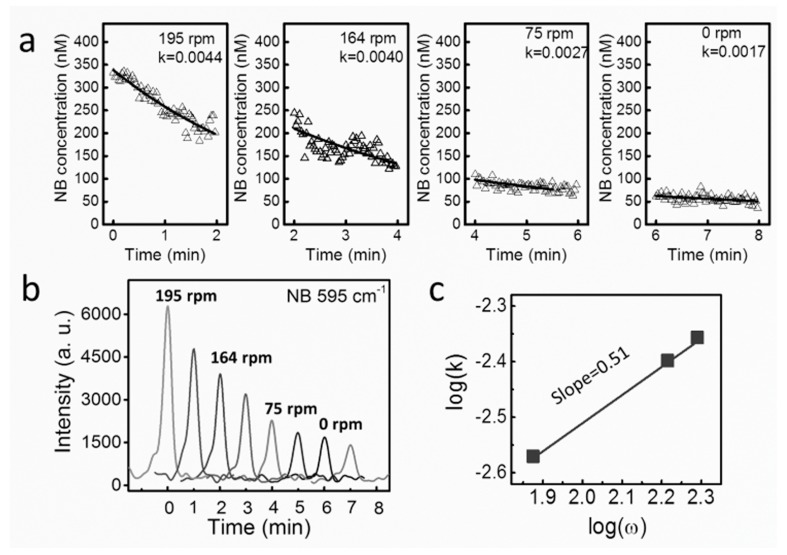
Tunable release of NB molecules by the mechanical rotation of nanomotor sensors. (**a**) Time dependent concentration of NB at various rotation speeds; (**b**) Raman spectra of R6G every 1 min at each rotation speed; (**c**) The power law dependence of molecule release rate versus rotation speed of nanomotors. With permission from [106].

By controlling the applied voltages, the plasmonic nanomotors were rotated at various speeds and demonstrated molecule release with tunable rate (Figure 16 and Figure 17) [106]. Nile blue (NB) and R-6G molecules were chosen for the demonstration due to their large Raman scattering cross-sections for facile Raman detection and wide usage in biomolecule tagging [20]. Experimentally, we detected the time-dependent release rate of either NB molecules (340 nM) or mixture of NB (~a few nM) and R-6G molecules (~20 µM) from single rotating nanomotors with SERS spectroscopy operating at a speed of 0.5 spectrum/s. 

**Figure 18:**
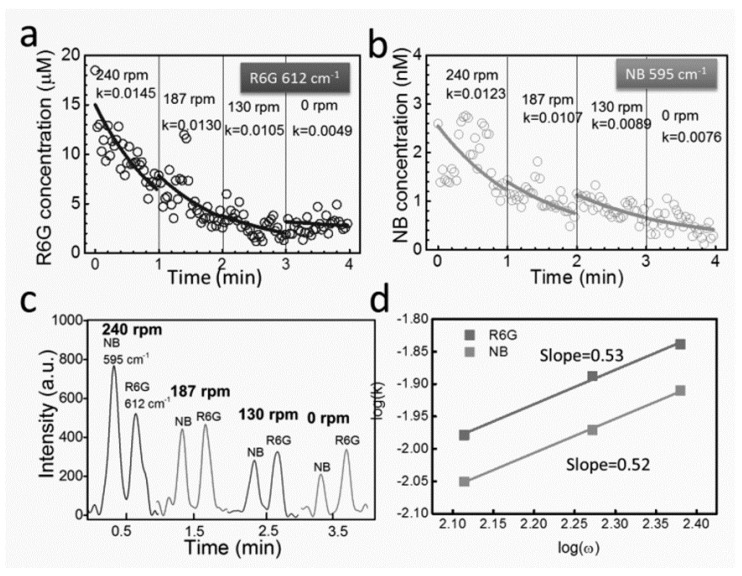
(**a**,**b**) Time dependent release of bi-analytes [NB (a) and R6G (b)] at different rotation speeds; (**c**) Raman spectra of NB and R6G every 1 min with different rotation speeds; (**d**) The power-law dependence of release rates of both analytes of NB and R6G versus the rotation speed. With permission from [106].

The concentration of molecules for either single or multiple molecular species decreases with time monotonically (Figure 17 and Figure 18). This is due to the net molecular diffusion from the nanomotor sensors to the bulk solution owing to the concentration differences and can be understood by using the Nernst diffusion-layer theory [107]. According to the theory, at the interface of a solid surface and a suspension medium, a stationary liquid layer with thickness of λ is formed. In such a layer, the transport of molecules between the solid surface and bulk liquid can only occur by the passive diffusion along the concentration gradient.

**Figure 19:**
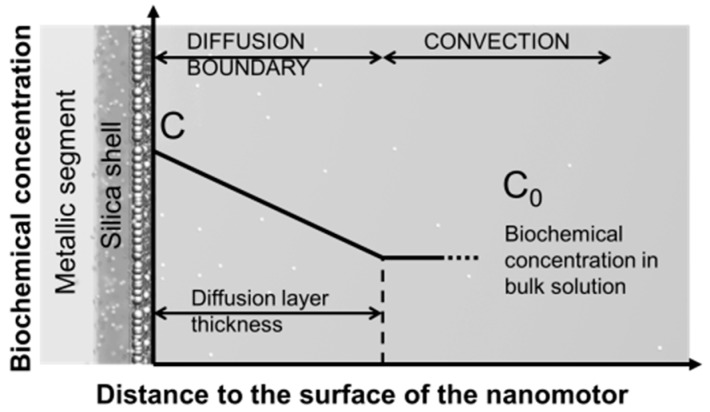
Schematic diagram of double layers next to the surface of the nanomotors. (small spheres: chemical molecules; big spheres: Ag NPs), with permission from [106].

Out of the region of the stationary layer, liquid convection dominates and molecules can be quickly transported and mixed in the bulk solution. Therefore, the concentration of the molecules of interest at the outer layer boundary is approximately same as that in the bulk solution. As a result, for molecules on the surface of nanomotors, a static concentration gradient is established across the diffusion layer (Figure 19). According to Fick’s laws, the concentration of molecules on the surface of nanomotors (*C*) is given by:

(2) $$C=C^{\prime }\times e^{-kt}+C_{0}$$

where $C^{\prime }=C_{1}-C_{0}$, $C_{1}$ is the initial concentration on the surface of nanomotors and *C_0_* is the concentration of molecules in the bulk liquid. The molecule release rate (*k*) is proportional to D/λ, the ratio of the diffusion coefficient of molecules (*D*) and the thickness of the static diffusion layer (λ). At different rotation speeds (*ω*), we can readily determine the corresponding value of *k* by fitting the experimental results with Equation (2) (Figure 17a,b and Figure 18a,b). It is found that the value of *k* monotonically increased with *ω* with a power-law dependence of *k* ~ *ω*^0.5^. This result is found universally for molecules of either single or multiple species releasing from nanomotors (Figure 17c). The mechanism is attributed to the thickness reduction of the stationary diffusion layer (λ), which is inversely proportional to the square root of the liquid convection speed, given by $\lambda \sim v^{-0.5}$ [108]. Also known that the molecular release rate of *k* ~ D/λ ~ 1/λ and the fluidic velocity *v ~ ω*. Therefore, it can be readily determine that $k\;\sim \;\sqrt{\omega }$. This analysis quantitatively agrees with our experimental observation of *k* ~ *ω^0.^*^5^ for both single and multiplex molecule release (Figure 17c and Figure 18d). Note that we carefully conducted a control experiment and confirmed the negligible effect of the AC electric field on the molecule release rate.

To the best of our knowledge, the previously discussed mechanism for tuning the molecule release by mechanical rotating nanomotors is the first of its kind. It is achieved from the unique motorized SERS nanosensors that can rotate while simultaneously detect with ultra-sensitivity. Note that molecules that can be tunably released as shown in the demonstration (Figure 17 and Figure 18) are not restricted to those Raman-sensitive species. It is applicable to any biochemicals of interest, such as drugs, cytokines [100], DNA molecules [109], peptides [110], antigens, enzymes [111], and antibodies, relevant to single cell stimulation, cell-cell communication, and system biology [100]. 

## 5. Summary

In summary, we have introduced the history and mechanisms of surface enhanced Raman scattering (SERS), discussed the state-of-the-art fabrication techniques of SERS substrates, and presented challenges in this field before reviewing our recent work on robotized SERS nanosensors for chemical detection. We rationally designed and fabricated SERS substrate to realize ultrasensitive and well reproducible molecule detection with an enhancement factor (EF) of ~10^10^, employed photonic devices and near-field effect to further boost the SERS enhancement, and adopted the electric and magnetic tweezers to motorize the SERS nanosensors for location predicable sensing and single live cell analysis. The assembling of the SERS nanosensors into rotary nanomotors enabled tunable molecule release and the time-dependent detection, which is the first of its kind. Overall, the research discussed in this work could open up many new opportunities in the field of biochemical detection, cell-cell communication, and drug delivery.
